# Children’s Informant Judgments and Recall of Valenced Facts at a Science Center

**DOI:** 10.3389/fpsyg.2021.659633

**Published:** 2021-06-16

**Authors:** Kimberly E. Marble, Jessica S. Caporaso, Kathleen M. Bettencourt, Janet J. Boseovski, Thanujeni Pathman, Stuart Marcovitch, Margaret L. Scales

**Affiliations:** ^1^Development and Understanding of Children’s Knowledge Lab, Department of Psychology, University of North Carolina at Greensboro, Greensboro, NC, United States; ^2^Memory Development Lab, Department of Psychology, York University, Toronto, ON, Canada; ^3^Department of Social Sciences and Health Policy, Wake Forest School of Medicine, Winston-Salem, NC, United States

**Keywords:** social cognition, expertise, positivity bias, memory, museum learning

## Abstract

In laboratory-based research, children recognize who is an expert and demonstrate an interest in learning from that person. However, children prefer positive information in the moment and sometimes prioritize positivity over expertise. To what extent do these social judgments (e.g., a preference for positivity) relate to information that children remember? We investigated the relation between these judgments and memory at a local science center to better understand children’s learning outcomes in naturalistic settings. We examined the extent to which 4- to 8-year-olds accepted facts about an unfamiliar animal from a zookeeper informant (i.e., expert) and a maternal figure (i.e., non-expert) when these facts were positive, negative, or neutral. Children endorsed positive information as correct, regardless of expertise, but demonstrated the strongest memory for neutral information. We discuss the implications of this dissociation for learning outcomes in naturalistic contexts as well as theoretical frameworks regarding children’s learning from others.

## Introduction

Children’s trips to science centers and museums promote educational interactions with parents and provide access to experts. Therefore, it is important to understand the factors that influence children’s perceptions of these individuals as sources of information. Indeed, children recognize both parents and experts as reliable (e.g., Kruglanski et al., 2005). During middle childhood, children are increasingly attentive to expertise (e.g., Danovitch and Keil, 2004), but sometimes disregard accurate information from knowledgeable people in favor of information that promotes a positive view of the world (i.e., positivity bias; Boseovski, 2010; Landrum et al., 2013). In some circumstances, the valence of information (i.e., positive or negative) also impacts children’s learning (e.g., acquisition of abstract words; Ponari et al., 2020) and emotional arousal or valence can impact visitors’ memories of science center exhibits (e.g., Falk and Gillespie, 2009). This influence of valence, coupled with children’s sensitivity to expertise, may shape children’s science center learning outcomes.

In the present study, we examined the extent to which expertise and valence influence children’s judgments of parents and experts as well as children’s memory for exhibit information. Children evaluated positive, negative, and neutral facts that a zookeeper informant (i.e., expert) and a maternal figure (i.e., non-expert) provided about a novel animal at a local science center. Children judged which individual was correct about the animal. We also examined children’s attributions of knowledge toward parents and experts for information that was unrelated to the animal (i.e., knowledge boundary judgments). Finally, we examined whether the information that children remembered about the animal was influenced by its valence or the expertise of the informant (i.e., source).

### Children’s Learning From Experts and Parents

We focused on children’s evaluation of parents and experts in the present study for several reasons. First, both parents and experts are readily available interaction partners in naturalistic science center settings (e.g., Pattison et al., 2017). Second, children demonstrate awareness of expertise but continue to prefer parents as sources of information even in domains where a parent lacks expertise (e.g., Raviv et al., 1990). By age 4, children distinguish experts from non-experts and understand that the expert is a better source of information (e.g., Koenig and Jaswal, 2011). During middle childhood, children build on this ability to evaluate whether an expert’s knowledge is relevant for a particular context (e.g., Danovitch and Keil, 2007). Despite young children’s sensitivity to expertise cues (e.g., labels such as “animal expert”; Taylor et al., 1994), many children view their parents as reliable sources of information about the world in general (Fonagy et al., 2007). Young children tend to trust a parent over a stranger (Corriveau et al., 2009), and between ages 4 and 10, children judge their parents to be trustworthy sources across several domains (e.g., social issues and school subjects; Raviv et al., 1990). In fact, children continue to view parents as knowledgeable despite experience with individuals who are more informed (e.g., a science teacher; Kruglanski et al., 2005). Finally, the contrast between parents and experts was of interest in the present study because parents and experts (e.g., zookeepers or science educators) influence children’s attitudes about wildlife through the transmission of positive and negative descriptions of animals (e.g., Reames and Rajecki, 1988; Muris et al., 2010).

Despite children’s perceptions of parent and expert knowledge, children’s acceptance of information from these individuals is influenced by its valence (see Marble and Boseovski, 2020). Children’s judgments of parents and experts may not coincide with their actual behavior when valence and expertise are salient. In one study, Boseovski and Thurman (2014) investigated whether 3- to 7-year-olds accepted positive and negative facts about an unfamiliar animal. The experimenter introduced a novel animal (e.g., a cuscus) with a few neutral facts and a photograph of the animal. Then, the experimenter displayed photographs of a zookeeper (i.e., expert) and a maternal figure (i.e., non-expert) and told children what each individual said about the animal. Half of the children heard a positive statement from the zookeeper (e.g., it is “friendly” and “loves playing with children”) and a negative statement from the maternal figure (e.g., it is “dirty and smelly” and “carries lots of germs”); this contingency was reversed for the other half of the children. Children were asked which person they thought was correct about the animal and were invited to “touch” the animal (unbeknownst to the children, it was a stuffed toy in an opaque crate). Three- to 5-year-olds accepted the expert’s statements as correct irrespective of whether she provided positive or negative facts but reached more readily into the crate when the maternal figure provided positive information about the animal. This finding highlights a dissociation between young children’s judgments and their actual behavior. In contrast and consistent with a positivity bias, 6- to 7-year-olds endorsed whichever source stated positive facts regardless of expertise. Older children were also more likely to reach into the crate when they endorsed positive information as correct. These findings from the older children indicate that 6- to 7-year-olds have difficulty accepting correct, negative information from qualified experts and may favor a non-expert in some contexts.

In addition to the influence of valence and source characteristics (e.g., expertise) on children’s judgments and behavior, valence and source characteristics (e.g., context and similarity) can also impact children’s memory (Foley, 2014; Van Bergen et al., 2015). In a science center context, judgments about the accuracy of an expert or a parent may operate as a notable source characteristic that biases children’s attention toward information from one of these sources (i.e., expert or parent) and increases memory for what that person says. In contrast, if children’s evaluation of correctness is a distinct process from any processes that facilitate recall, perhaps source characteristics such as expertise, would have less influence on memory performance relative to the valence of the information. The examination of this relation during real-time learning may inform how children’s beliefs about, and behavior toward, wildlife develop. Therefore, it is important to extend this paradigm to a naturalistic setting that involves live informants.

In everyday situations, children may socialize with adults who do not fit neatly into a single category. These real-world categorizations may influence children’s inferences about what parents and experts know, which in turn might affect who children endorse as correct during learning experiences. For example, some parents hold a dual role as both a caregiver and an expert in a separate domain. Children who are aware of this context may use it to compare knowledge between adults with overlapping roles (e.g., a zookeeper who is also a parent) or to judge whether an individual is knowledgeable across multiple domains. In contrast, many parents may not have expertise relevant to a science center setting. In this case, boundaries between parent and expert roles should inform children’s evaluation of each individual’s knowledge in a science center setting. Children who are not sensitive to these differences might overgeneralize what parents and experts know. Indeed, developmental differences in children’s reasoning about categorical hierarchies might influence these knowledge judgments (Blewitt, 1994).

Beginning in the preschool period, children make some inferences about an individual’s behavior and mental states according to that person’s membership in a particular category (e.g., a gender category; Rhodes et al., 2014). With regard to children’s judgments about expertise, children may use occupation information to make category-level inferences about what zookeepers know (in general) compared to what parents know (in general). The salience of these potential knowledge differences between experts and parents might be amplified in a science center context. In addition, there is age-related improvement in children’s understanding of appropriate generalizations concerning what an expert knows outside of his or her domain of expertise (e.g., Taylor et al., 1994; Keil et al., 2008; Danovitch and Noles, 2014). If children’s reasoning about boundaries to parent knowledge follows a similar pattern to children’s reasoning about boundaries to expertise, children might be most likely to rely on an expert to reconcile conflicting information provided by the expert versus a parent. However, science centers also promote informal learning with parents (e.g., Callanan et al., 2017), which in turn may promote the integration of information shared by both zookeepers and parents into children’s knowledge.

### Children’s Memory in Science Center and Museum Settings

The social context provided by parents and experts in science centers may impact children’s memory for those experiences. Children may weigh what parents and experts say (i.e., content) in these settings against beliefs about whether parents and experts are qualified sources of information (i.e., knowledgeable) in science center contexts. Indeed, parent and museum staff facilitation of children’s engagement and learning in science center contexts is of strong practical interest to museum educators (e.g., Pattison et al., 2017). In recent research, interactions between parents and children during exhibit exploration have been a focal point (Benjamin et al., 2010; Jant et al., 2014). Parents’ conversation style is one factor that is related to children’s memory for events (Nelson and Fivush, 2004; Fivush et al., 2006). In museum contexts, children whose parents asked more open-ended *Wh*-questions during exhibit conversations remembered more about the experience later that day and after a 2-week delay (Benjamin et al., 2010; Jant et al., 2014). In this way, children’s conversations with parents can provide social support for learning at exhibits. With age, children recall increasing amounts of event detail, need fewer cues to recall an event and are better able to discern when some types of cues are helpful, and demonstrate an improved ability to remember events after longer delays (Bauer, 2007; Reese et al., 2011; Selmeczy and Ghetti, 2019). Taken together with the important role that parents have in museum-based conversations and learning, these age-related changes may have an important effect on children’s memory for exhibit information.

Specifically, age-related improvements in source memory, defined as memory for perceptual and contextual information of an event (Johnson et al., 1993), might be particularly important in a science center context. The ability to remember the source of information improves between ages four and seven (Drummey and Newcombe, 2002; Riggins, 2014; for review, see Foley, 2014) and may facilitate recall of factual information (Bemis et al., 2013). In science center settings, children encounter a variety of sources, including both parents and experts. Similarity between sources (e.g., appearance of a person and type of information shared) may make it difficult for children to attribute accurate source information (e.g., Lindsay et al., 1991). Salient differences between sources (e.g., expertise level and information valence) may help children distinguish between the sources and organize facts to facilitate later recall, especially if these differences highlight familiar categories (e.g., experts vs. non-experts) or align with preexisting learning preferences (e.g., bias toward positive information). Given the age-related improvements in source memory, older children may be more likely than preschoolers to take advantage of these cues during recall, but to our knowledge, there has been no research on the effect of source expertise on memory in these settings.

In addition to children’s ability to leverage source information, children’s strengthening preference for positivity during middle childhood (Boseovski, 2010) may explain mixed findings regarding developmental differences in the effect of valenced content on children’s memory. For example, children sometimes demonstrate better memory for negative information (e.g., threatening social behavior, Baltazar et al., 2012; traumatic events, Pezdek and Taylor, 2002), but in other circumstances, children demonstrate a bias for positive information during recall (e.g., word list; Brainerd et al., 2010). It is possible that in certain situations, valenced information is salient overall and remembered better relative to neutral information. Indeed, children remember positive and negative personal events equally well most of the time (Fivush, 1998) and both positive valence and negative valence help children acquire abstract concept words (e.g., Ponari et al., 2018). Young children may be sensitive to emotionally salient content about people or animals, regardless of the valence of that content. In one study, 4- to 6-year-olds heard several stories about animal characters that experienced a positive, negative, or neutral event. One hour later, children were asked what they could recall from the stories. Children’s memory was better for positive and negative contents relative to neutral content, but best for negative content overall (Van Bergen et al., 2015). It may be beneficial for children to remember negative messages that contain safety warnings, threats to self, or threats to animals when learning about wildlife (Boseovski and Thurman, 2014; Burris et al., 2019) and yet 6- to 7-year-olds have demonstrated a bias for positive information about animals (Boseovski and Thurman, 2014). Indeed, 7- and 11-year-olds recall positive and neutral words better than negative words in laboratory-based, list recall tasks (e.g., Howe et al., 2010) and 8- to 9-year-olds are more accurate when tested for their acquisition of novel abstract words that are positive relative to neutral (Ponari et al., 2020).

This pattern of age-related increase in recall of positive content aligns with a general developmental trend to endorse positive feedback and positive testimony from others (Marble and Boseovski, 2020). Taken together, these findings across literatures may suggest that children’s judgments about informant sources could influence children’s memory for the information those sources provide. Both positive information and negative information might be salient for recall: Positive information aligns with a strengthening preference for positivity, whereas negative information violates this preference. Another possibility is that the mixed findings regarding how valence influences memory indicate a dissociation between children’s correctness judgments and the processes that influence children’s memory. The relation between valence, expertise, and memory is particularly important for children’s learning in science center settings given that children’s early positive or negative experiences with animals are thought to lay the foundation for attitudes toward wildlife later in life (Kidd and Kidd, 1996).

### Current Study

We examined whether 4- to 8-year-olds’ acceptance of information about an unfamiliar animal differed based on the expertise of the informant (i.e., zookeeper vs. non-expert maternal figure) and the valence of the informants’ statements (i.e., positive, neutral, or negative). We extended the paradigm used by Boseovski and Thurman (2014) in two ways. First, we adapted the paradigm to examine the effect of “live” experts and non-experts in a naturalistic setting (i.e., a local science center). Second, we included a memory assessment to examine whether the effect of expertise and valence on children’s learning of information was similar or distinct from the effect on judgments of source correctness (i.e., correctness judgments). Consistent with a strengthening positivity bias across middle childhood (Boseovski, 2010; see Boseovski and Thurman, 2014), we anticipated that 4- to 5-year-olds might accept more of the expert’s facts regardless of valence relative to 6- to 8-year-olds. We predicted that 6- to 8-year-olds would perform better than 4- to 5-year-olds when asked to infer other types of knowledge for the informants (i.e., knowledge boundary judgments) and that these older children would recall more facts (memory assessment). We did not have specific predictions regarding the interaction of valence and expertise on recall performance, given evidence that both positive and negative experiences are remembered (Wolins et al., 1992; Kidd and Kidd, 1996).

## Materials and Methods

### Participants

Eighty 4- to 8-year-olds (*M* = 77.36 months, *SD* = 17.30 months; 36 girls) were recruited from the local science center or a database of volunteers from the community. This sample size was estimated based on the paradigm adapted from Boseovski and Thurman (2014) that produced between a medium and large effect for similar main measures ($\eta_{p}{}^{2}$ = 0.14). Demographics of the sample reflected the overall visitor demographics of the local science center where testing took place. With regard to participant race, 77.5% reported this information and these parents identified their children as White (80.6%), Black (9.7%), Asian (1.6%), or bi-racial (8.1%).

With regard to annual household income, 71.3% reported this information and the majority of these household incomes were above the city average at the time of data collection (42.1% reported above $90,000; 22.8% as $60,000–$90,000). Families who approached the indoor aquarium and animal exhibits were asked whether they would like to participate in a research opportunity and were provided with basic information about the exhibit of interest. Parents provided written consent for their children’s participation, and children 7 years of age and older provided written assent. Approval for this study was obtained from the university’s institutional review board, and a memorandum with the science center was completed.

### Materials

The informants were trained researchers playing the roles of a zookeeper and a maternal figure. There were four total researchers who were trained for this role, but only two researchers acted in these roles per participant (i.e., one zookeeper and one maternal figure). The zookeeper informant wore a black polo shirt with khaki pants and carried a clipboard and a walkie-talkie. The maternal figure informant wore a black blouse with jeans and carried a purse and a map of the science center. The informants provided information about the tamandua, a species of anteater native to Central and South America that was on exhibit at the science center.

### Design

A 2 (age: 4- to 5-year-olds vs. 6- to 8-year-olds) × 3 (fact valence: positive, neutral, and negative) × 2 (informant status: zookeeper expert vs. maternal figure non-expert) mixed design was used with fact valence and informant status as the within-subjects factors.

### Procedure

After consent was obtained, the experimenter escorted participants to the exhibit that housed the tamandua. During this time, the experimenter confirmed that participants had no prior knowledge of tamanduas. At the exhibit, the experimenter said, “This is Jess, and this is Kim. They want to tell you what they know about the tamandua.” The zookeeper introduced herself with the statement: “I am a zookeeper. I work with many different kinds of animals. I know a lot about all kinds of animals that most other people don’t know about.” The maternal figure introduced herself with the statement: “I am a mom just like any regular mom. I have two kids around your age. I know a lot about being a mom, just like any regular mom does” (adapted from Boseovski and Thurman, 2014). The order of introductions was counterbalanced. The informant role played by each researcher was counterbalanced, and half of the participants were introduced to “Jess the zookeeper and Kim the mom” and the other half were introduced to “Kim the zookeeper and Jess the mom.”

Next, the experimenter said, “Now Jess the zookeeper is going to tell you what she knows about the tamandua.” The informant guided participants closer to the window of the exhibit. Thus, participants had a “live” view of the animal, which was typically sleeping and partially obscured in a leaf-covered area of the exhibit. The informant pointed out the tamandua and presented her facts about the animal to participants (e.g., “Tamanduas live in tropical rain forests”; see Table 1 for full scripts). The other informant stood out of earshot. After the first informant finished telling participants everything she knew about the tamandua, the second informant approached the participants to present her facts about the tamandua. The order in which informants shared facts and the script assigned to each informant were counterbalanced across participants.

**Table 1:** Full scripts, sorted by valence and conflicting facts, for each informant.

Script A: Non-conflicting facts	Script B: Non-conflicting facts
**Positive**	
Baby tamanduas are cute and cuddly	Tamanduas have a great sense of smell
They are good climbers	They have strong arms and legs
Tamanduas also have really good hearing and hear from far away	Mother tamanduas take good care of their babies and give them piggy back rides
**Negative**	
Brother and sister tamanduas do not get along and push and fight each other	Tamanduas also have bad vision and cannot see far away
They are smellier than a skunk	Adult tamanduas are slow and lazy
They have long, sharp claws	They are bad runners
**Neutral**	
They are nocturnal, meaning they are awake at night	Their fur can be many colors
They live in nests on the ground	They live in a tropical rain forest
Babies do not look like parents	Tamanduas can swim in lakes and rivers
**Script A: Conflicting facts**	**Script B: Conflicting facts**
**Positive in Script A conflicts with negative in Script B**	
Tamanduas are gentle and purr softly	They are mean and roar loudly
Tamanduas love to live in homes as pets	Tamanduas hate to live in homes as pets
They also have big brains and remember a lot	Tamanduas have small brains and forget often
**Negative in Script A conflicts with positive in Script B**	
Tamanduas are very dirty and carry germs	They are very clean and healthy
Tamanduas fight a lot with other animals	They are very friendly with other animals
They have a hard, scaly tail that they use to break things around them	They also have a soft, furry tail that they use as a pillow to sleep
**Neutral**	
Other than zoos, they only live in Argentina	Other than zoos, tamanduas only live in Brazil
Their favorite food is termites	Their favorite food is beetles
Also, their babies are born with their eyes closed	Their babies are born with their eyes open

The informant scripts (Script A and Script B) each consisted of 18 facts about the tamandua. Twenty-four randomized versions of each script were used. Each script contained: six positive facts (e.g., “Baby tamanduas are cute and cuddly”), six negative facts (e.g., “Tamanduas are smellier than a skunk”), and six neutral facts (e.g., “Tamanduas can swim in lakes and rivers”). Nine of the facts in a script, three of each valence, conflicted with the nine facts on the same topics in the other script. For example, one informant told the participants “Tamanduas are mean and roar loudly” but the other informant told the participants “Tamanduas are gentle and purr softly” (adapted from Boseovski and Thurman, 2014; see Table 1).

**Figure 1:**
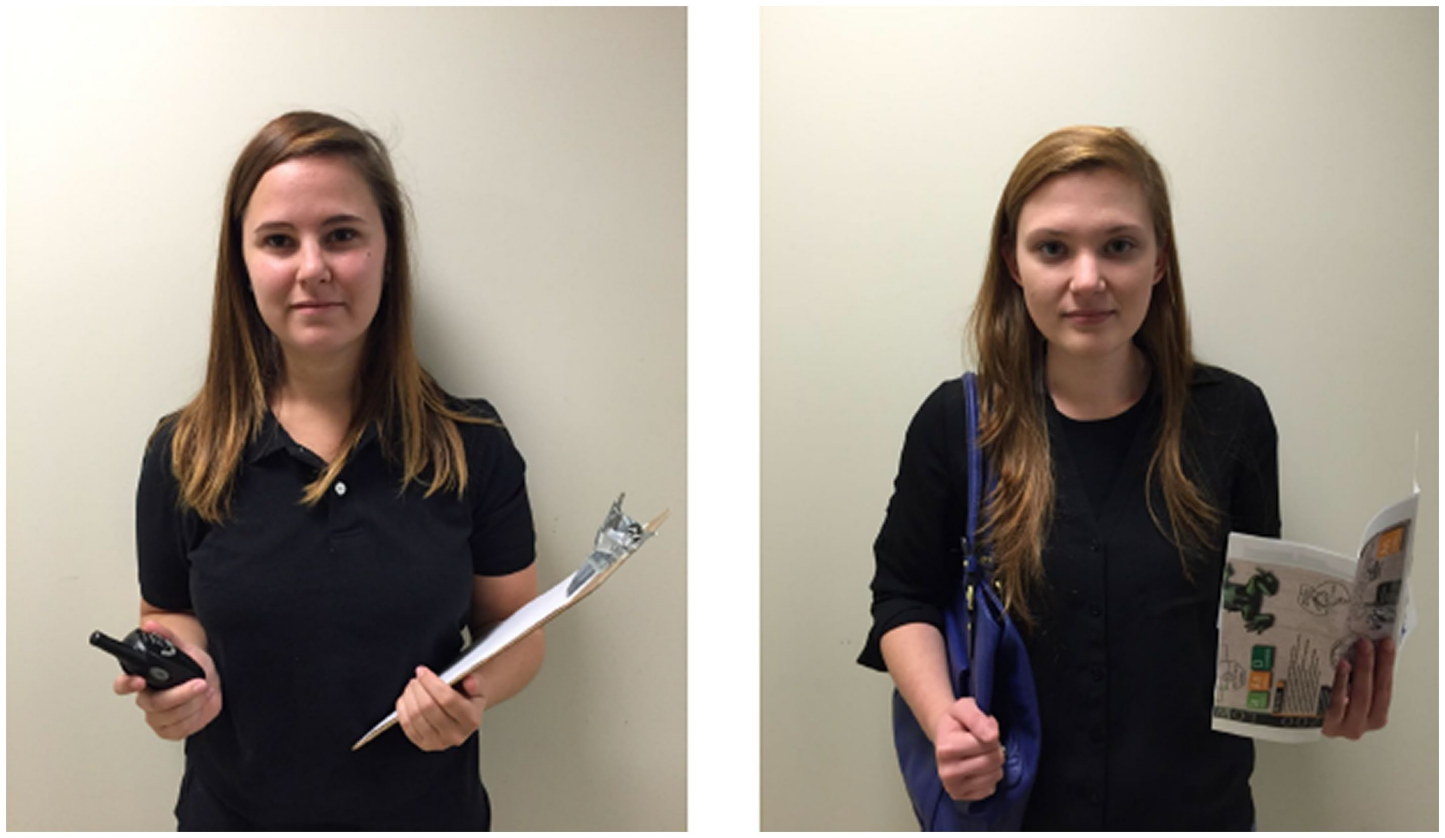
Sample photographs of “Jess the zookeeper” and “Kim the mom,” the trained researchers who acted in informant roles for the purpose of the study.

After each informant spoke to the participants, the experimenter escorted the participants to a private room nearby to complete three assessments, described below (correctness judgments, knowledge boundary judgments, and a memory assessment). The correctness judgments and knowledge boundary judgments were conceptualized as two parts of a social cognition task. The order in which this social cognition set versus the memory assessment was administered was counterbalanced across participants, and participants’ responses were recorded on an iPad by the experimenter. Photographs of the informants were displayed as a reference for the participants during these assessments (see Figure 1).

#### Correctness Judgments

These items evaluated how children judged the correctness of conflicting facts presented by the zookeeper informant and the maternal figure. The questions pertained to the nine conflicting facts presented by the informants (see Table 1). Participants were shown the photographs of each informant and reminded which informant told them each of these nine facts (e.g., “Jess the zookeeper said that tamanduas are gentle and purr softly but Kim the mom said they are mean and roar loudly”). Then, participants were asked a forced-choice question “Who do you think is right?” (answer options: zookeeper or maternal figure). Participants’ responses were summed across each combination of informant status and fact valence to reflect the number of times participants endorsed the zookeeper when she presented a positive fact, when she presented a negative fact, and when she presented a neutral fact; and the number of times participants endorsed the maternal figure when she presented a positive fact, when she presented a negative fact, and when she presented a neutral fact. For example, if a participant endorsed all three positive facts presented by the maternal figure, that participant would receive a “3” for the maternal-positive fact set but that would mean that the same participant endorsed zero negative facts presented by the zookeeper informant and would receive a “0” for the zookeeper-negative fact set (see Table 1). Collapsed across informant status, participants’ responses could be summed out of a possible total of six valence-specific endorsements (i.e., endorsement of positive, neutral, or negative facts). Collapsed across fact valence, participants’ responses could be summed out of a possible total of nine informant-specific endorsements (e.g., a participant who endorsed the zookeeper informant for all the conflicting facts would receive a “9” for zookeeper correctness judgments but “0” for maternal figure correctness judgments).

#### Knowledge Boundary Judgments

This assessment evaluated children’s understanding of the boundaries of expertise and consisted of 17 questions, which served as a supplemental measure to examine whether children extended informant knowledge beyond knowledge of tamanduas; the valence of these facts was not manipulated, and items were randomized across subsets during presentation. There were three subsets of questions: four questions about topics most related to a zookeeper’s expertise (e.g., “Who knows more about why fish live in water?”), four questions about topics most related to a mother’s knowledge (e.g., “Who knows more about how to strap in a car seat?”), and nine questions about topics that reflect general knowledge (e.g., “Who knows more about why we tell the truth?”). In each set, there were three answer choices (zookeeper informant, maternal figure, or both informants would know about the topic) and participants received a score of 1 for each question where they indicated the expected choice (i.e., “zookeeper” for the zookeeper subset, “mom” for the mother’s knowledge subset, and “both” for the general knowledge items). All other answers received a score of 0. Previous studies regarding children’s inferences about knowledge related to biological and social psychology principles were consulted to inform the creation of these items (e.g., Danovitch and Keil, 2004, 2007). In addition, the research team members who created these items obtained informal feedback from other members of the laboratory regarding how reasonable it would be to expect most adults to know some of these items to justify the expected answer choice of “both” and informal feedback regarding knowledge that would be specific to mothers/parents. Participants’ responses were summed for each subset to produce three scores (out of 4 points, 4 points, and 9 points, respectively).

#### Memory Assessment

This assessment included an open-ended free recall prompt [“You heard information about tamanduas from (Informant 1) and (Informant 2). What did you learn about the tamandua?”] followed by six, topic-based cued recall questions (e.g., “Now I’m going to ask some questions, some of which you already talked about. Just answer them the best that you can. Okay, ready? What do you remember about where tamanduas live? What do you remember about what tamanduas look like? What do you remember about what tamanduas are good or bad at?”). Participants could recall information for up to 36 facts (18 facts per informant, divided equally across valence, and including conflicting facts). Participants could receive points for recalling a fact that was stated by the zookeeper, the maternal figure, or recalling what both informants said. In addition, participants could respond with more than one fact to address each cued recall question to maximize reports of any information that children could remember. For example, the question “What do you remember about how tamanduas act?” could be answered by recalling information that informants provided about interactions with other animals and/or information provided about how tamanduas sleep (i.e., not participants’ observations while at the exhibit).

Participants were scored based on the amount of accurate detail that they provided about a recalled fact to provide the most generous scoring for the youngest participants (4-year-olds), who might only be able to recall partial facts or partial details or might only be able to report partial facts due to language ability. We adapted a scoring scheme from the vocabulary section of the Wechsler Abbreviated Scale of Intelligence (WASI-II; Wechsler, 2011). Participants could receive a total score out of a possible 72 points: Participants received 2 points for each fact that they remembered fully (i.e., complete detail from the informant’s statement); participants received 1 point if they remembered the general statement accurately but without full detail. For example, one informant stated that tamanduas eat beetles. For the question “What do you remember about what tamanduas eat?” a participant who responded “beetles” received 2 points, but a participant who responded “bugs” received 1 point for providing generally correct information. Participants did not receive any points if they gave incorrect statements (i.e., information that did not resemble either informant’s statement) or unrelated filler statements (e.g., “I don’t know”). One member of the research team scored all these responses, and a second research assistant scored 50% of these responses. Interrater agreement for classifying these responses was strong, 1-point responses: ICC (2,2) = 0.995; 2-point responses: ICC (2,2) = 0.963. Disagreements were resolved by a third member of the research team.

After testing was complete, participants were debriefed. They were told that the informants were not really a zookeeper or a mom and the experimenter ensured that children understood the informants had been “pretending” just for that day. The experimenter also made sure that children and their parents knew that some of the facts that they heard about the tamandua were inaccurate. Families were provided with a fact sheet created by the museum’s education team that contained accurate information about the tamandua.

## Results

### Correctness Judgments

**Figure 2:**
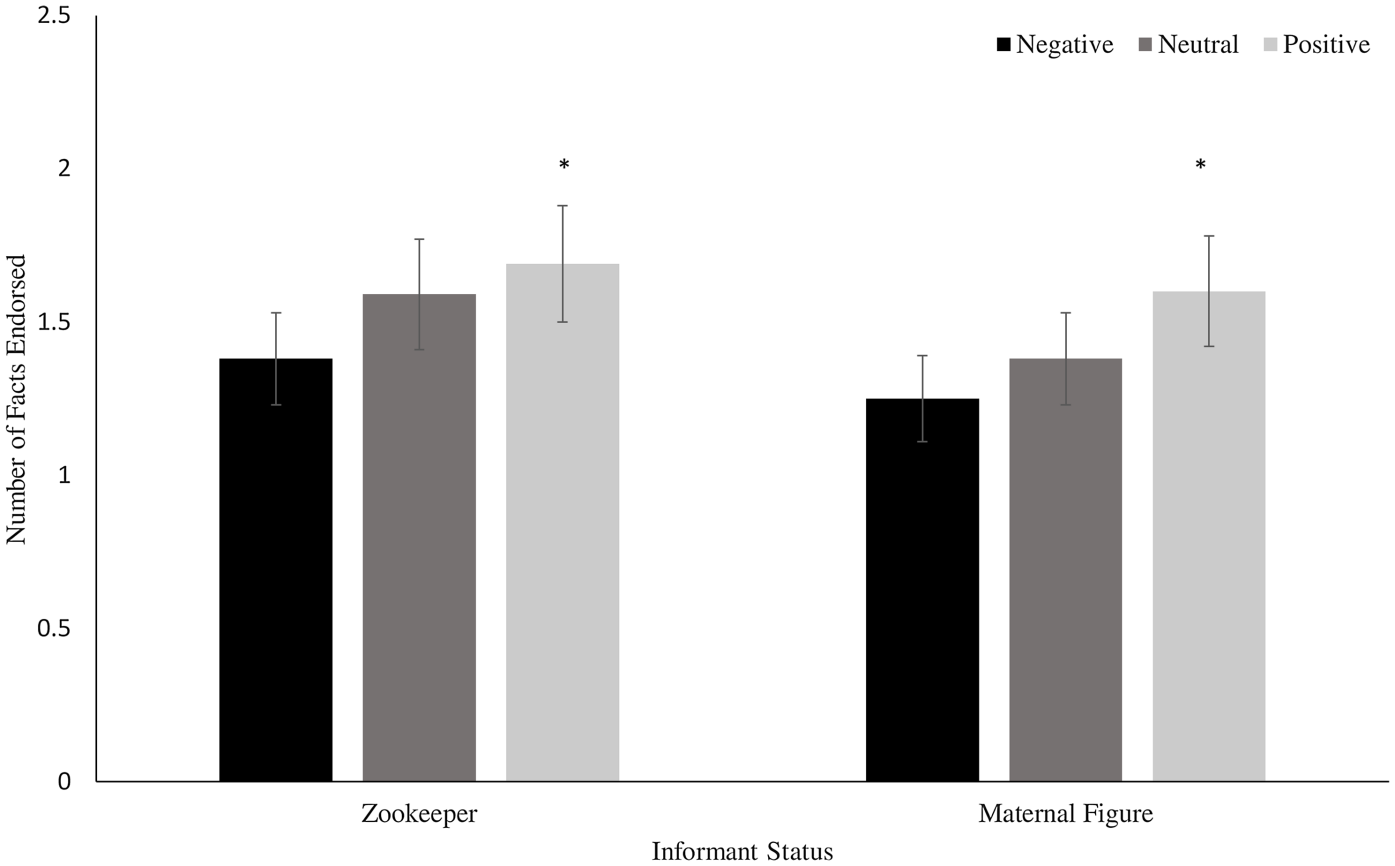
Mean number of facts endorsed as correct by informant status and fact valence. ^*^indicates significantly different from both negative and neutral facts, *p* < 0.05. Error bars reflect standard errors.

A 2 (age group, between subjects) × 3 (valence, within subjects) × 2 (informant status, within subjects) mixed ANOVA conducted on the correctness score revealed a main effect of valence, *F*(2, 78) = 4.06, *p* = 0.02, $\eta_{p}{}^{2}$ = 0.095. Children endorsed positive facts (*M* = 3.29, *SD* = 1.12) as correct over neutral facts (*M* = 2.96, *SD* = 0.25) which in turn were endorsed over negative facts (*M* = 2.63, *SD* = 1.14; all Bonferroni-corrected pairwise comparisons *p*s < 0.01; *d*s = 0.29, 0.30, and 0.31; see Figure 2). There were no main effects of informant status or age group and no significant interactions among these factors (all *p*s *>* 0.30).

### Knowledge Boundary Judgments

The data from 10 participants were not included as they did not receive the option to select “both” informants due to experimenter error. The data for the remaining 70 participants (26 4- to 5-year-olds) were analyzed using a one-way ANOVA to compare the effect of age group on each of the generalization of knowledge scores (i.e., “zookeeper,” “mother,” and “both” knowledge areas).

For the “zookeeper” item set, there was a significant effect of age, *F*(1, 68) = 13.84, *p* < 0.001, $\eta_{p}{}^{2}$ = 0.17. Older children (*M* = 3.11, *SD* = 1.03) selected the zookeeper as knowledgeable more often than younger children (*M* = 2.08, *SD* = 1.18). To examine whether this effect indicated that only older children selected the zookeeper systematically, *t*-tests against chance (2 out of 4) were conducted. Older children selected the zookeeper at a rate significantly different from chance, *t*(43) = 7.11, *p* < 0.001, *d* = 1.04; younger children were unsystematic, *t*(25) = 0.31, *p* = 0.76.

For the “mother” item set, there was a significant effect of age, *F*(1, 68) = 18.30, *p* < 0.001, $\eta_{p}{}^{2}$ = 0.21. Older children (*M* = 3.18, *SD* = 1.15) selected the maternal figure as knowledgeable more often than younger children (*M* = 1.77, *SD* = 1.53). *T*-tests against chance (2 out of 4) revealed that older children selected the maternal figure at a rate significantly different from chance, *t*(43) = 6.61, *p* < 0.001, *d* = 0.99; younger children were unsystematic, *t*(25) = −0.76, *p* = 0.46.

Finally, for the “general” item set, there was a significant effect of age, *F*(1, 68) = 4.24, *p* = 0.04, $\eta_{p}{}^{2}$ = 0.06, such that younger children (*M* = 3.77, *SD* = 2.95) selected “both” informants as knowledgeable more often than older children (*M* = 2.45, *SD* = 2.42). *T*-tests against chance (3 out of 9) revealed that neither older nor younger children selected the expected answer of “both” at a rate significantly different from chance: older, *t*(43) = −1.49, *p* = 0.14; younger, *t*(25) = 1.39, *p* = 0.18. Additional *t*-tests against chance to examine whether children favored either informant revealed that older children selected the maternal figure systematically, *t*(43) = 6.11, *p* < 0.001, *d* = 0.92, and systematically refrained from selecting the zookeeper, *t*(43) = −8.36, *p* < 0.001, *d* = 1.26. Younger children also systematically refrained from selecting the zookeeper *t*(25) = −3.92, *p* < 0.001, *d* = 0.77, but younger children did not select the maternal figure at a rate significantly different from chance, *t*(25) = 0.69, *p* = 0.50. All of the older children and 20 out of 26 younger children (76.9%) endorsed the maternal informant for at least 5 of these 9 general knowledge items.

### Memory Assessment

Preliminary analyses indicated that there was no significant effect of assessment order (i.e., memory assessment first vs. correctness judgments first) on children’s recall of information about the tamandua, *F*(1, 79) = 2.39, *p* = 0.126. On average, children remembered approximately five facts about the tamandua (*M* = 5.20, *SD* = 2.84) out of 36 total facts. This average recall was not meaningfully increased when recall was summed across free and cued recall responses (*M* = 5.19). Therefore, the results reported below focus on cued recall only.

A 2 (age group, between subjects) × 3 (valence, within subjects) × 2 (informant status, within subjects) mixed ANOVA revealed a main effect of age, *F*(1, 78) = 18.71, *p* < 0.001, $\eta_{p}{}^{2}$ = 0.193, such that 6- to 8-year-olds (*M* = 8.67, *SD* = 3.92) remembered more information than 4- to 5-year-olds (*M* = 4.87, *SD* = 3.66).

**Figure 3:**
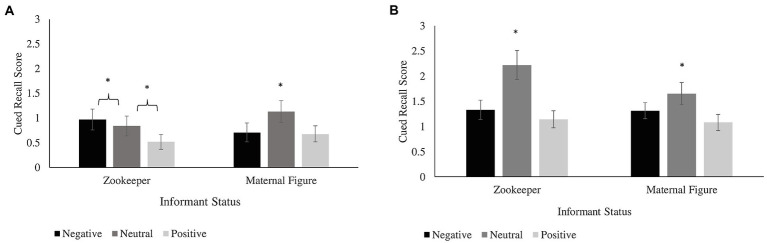
Cued recall score by informant status and fact valence for **(A)** 4- to 5-year-olds and **(B)** 6- to 8-year-olds. ^*^indicates significantly different from both negative and positive facts, *p* < 0.05. Error bars reflect standard errors.

There was also a main effect of valence, *F*(2, 78) = 12.79, *p* < 0.001, $\eta_{p}{}^{2}$ = 0.141. Children remembered more neutral information (*M* = 3.14, *SD* = 2.29) than positive information (*M* = 1.83, *SD* = 1.61) or negative information (*M* = 2.26, *SD* = 1.73; Bonferroni-corrected pairwise comparisons, *p* < 0.001, *d* = 0.52 and *p* = 0.002, *d* = 0.36); positive and negative information did not differ from one another (Bonferroni-corrected pairwise comparison *p* = 0.052). There were no significant interactions between valence, age, and informant status (all *p*s > 0.20); children remembered more neutral information irrespective of informant status or age (see Figure 3).

To investigate whether the main effect of age was due to the level of detail children remembered, a chi-square test of independence was conducted with the variables age group and level of detail (i.e., number of 1-point vs. 2-point responses). The relation between these variables was not significant, *χ*^2^(1, 80) < 0.000, *p* = 1.0. Descriptively, more children provided at least one 1-point response (90.32% of younger children and 93.88% of older children) relative to those who provided at least one 2-point response (67.74% of younger children and 91.84% of older children).

In addition to these analyses, children’s recall of the subset of conflicting facts was examined separately. On average, children recalled between 2 and 3 of the 9 conflicting facts (*M* = 2.76, *SD* = 1.74). A 2 (age group, between subjects) × 3 (valence, within subjects) × 2 (informant status, within subjects) mixed ANOVA was conducted on children’s conflicting fact cued recall score and revealed a similar pattern to children’s cued recall score out of all 36 facts. The analysis revealed a main effect of age, *F*(1, 78) = 8.99, *p* = 0.004, $\eta_{p}{}^{2}$ = 0.10, such that 6- to 8-year-olds (*M* = 3.20, *SD* = 1.71) recalled more conflicting information than 4- to 5-year-olds (*M* = 1.06, *SD* = 1.57). There was also a main effect of valence, *F*(1, 78) = 9.98, *p* < 0.001, $\eta_{p}{}^{2}$ = 0.11. Children remembered more neutral conflicting information (*M* = 1.34, *SD* = 1.11) than positive conflicting (*M* = 0.70, *SD* = 0.85) or negative conflicting information (*M* = 0.73, *SD* = 0.89; Bonferroni-corrected pairwise comparisons both *p*s < 0.001, *d*s = 0.41 and 0.42); positive and negative conflicting information did not differ from one another (Bonferroni-corrected pairwise comparison *p* = 1.0).

Finally, separate two-tailed Pearson correlations were conducted to examine children’s performance on the memory assessment in relation to their correctness judgments. Children’s cued recall for positive, neutral, and negative information was not related to their endorsement of positive, neutral, or negative information from either informant on the correctness judgments (all *p*s > 0.10). This pattern held when children’s cued recall for conflicting facts alone was examined separately by informant and when collapsed across informant (all *p*s > 0.10).

## Discussion

As predicted, children judged positive information about an unfamiliar animal as more correct, regardless of the expertise of the informant providing that information. Despite this preference for positive information on the correctness judgments, older children recognized expertise and inferred knowledge more accurately than younger children on the knowledge boundary judgments, which did not involve valenced information. Overall, children’s memory for the facts was relatively low, but our results were consistent with general age-related improvements: older children remembered more facts than younger children on the memory assessment. In contrast to children’s correctness judgments of conflicting testimony, when children recalled facts from the exhibit interaction, their memory was best for neutral facts. Taken together with older children’s performance on the knowledge boundary judgments, the findings from the memory assessment suggest that age-related improvements in children’s ability to identify who is a qualified source of information may not align with what children remember. We discuss the theoretical implications of this dissociation, along with the implications for children’s science center learning outcomes.

A central aim of this study was to examine the relation between children’s preference for positivity when they evaluate informants (e.g., correctness judgments) and what children remember in a naturalistic setting (e.g., memory assessment). In general, children’s recall of exhibit facts was low relative to the total amount of possible information that they could recall, and recall was not scaffolded by the presence of source expertise or valence. Children’s recall was unrelated to their judgments about which informant was correct (i.e., whichever informant presented positive facts). In contrast, when children remembered information about the tamandua, it was neutral rather than positive or negative information. In general, positive and negative information tend to be salient when children recall personal experiences (e.g., Fivush, 1998) or other narrative material (e.g., Potts et al., 1986), and this valenced information is often reported in children’s qualitative accounts of museum field trips (e.g., Wolins et al., 1992). It is surprising that the physical presence of the tamandua in this study did not heighten the salience of valenced content regarding its behavior (e.g., is this animal “friendly” or potentially aggressive?). Instead, children’s better recall of neutral relative to valenced information may indicate that neutral information was easier for children to process and remember considering potential distractions in a science center environment (e.g., other visitors and noise). However, we interpret children’s recall of neutral information with caution given the relatively small practical differences in recall across valence.

Somewhat surprisingly, children did not draw on source expertise to scaffold recall on the memory assessment. Older children could have used source expertise as a cue to recall accurate information given older children’s sensitivity to qualitative differences in the types of knowledge that others possess (e.g., Raviv et al., 1990; Danovitch and Keil, 2007). Specifically, it would be feasible for older children to demonstrate sensitivity to expertise during recall even if they demonstrated a preference for positivity when they evaluate the accuracy of sources for correctness judgments. However, source expertise may not have been salient enough in this study to elicit additional processing. Although the informants offered conflicting facts about the tamandua, each discussed the same aspects of the tamandua overall (e.g., habitat, behavior, and eating habits). In this way, the two informants may have presented an overall similarity to one another (e.g., Thierry and Pipe, 2009). If cognitive demands were high due to the number of potential cues and the amount of information presented, children may have been unable to use source expertise to scaffold recall.

Children also did not use expertise information to make correctness judgments about which informant they thought provided accurate information about the tamandua, but rather preferred positive statements. Despite children’s sensitivity to expertise across a variety of laboratory-based studies (e.g., Lane and Harris, 2015; Toyama, 2017), this prioritization of positive information is consistent with a sizable literature in which children’s correctness judgments or evaluation of expertise is influenced by valenced information (see Marble and Boseovski, 2020). This consistency with laboratory-based research suggests that the physical learning environment may not play a major role in children’s informant judgments. Instead, the ecological validity of laboratory-based selective trust studies might be strengthened by incorporating multiple or conflicting cues to knowledge. Theoretically, this correctness judgment finding suggests that children’s preference for positivity may be a motivation or belief-based bias, distinct from memory-related biases. Older children’s use of expertise information in the knowledge boundary judgments, in which the valence of information was not manipulated, supports this view. Older children demonstrated a nuanced ability to infer knowledge for the zookeeper informant and the maternal figure for the “zookeeper” and “mother” sets of this knowledge boundary task, respectively. This knowledge boundary judgment performance suggests that in the absence of valence information, older children may capitalize on other cues to evaluate testimony, including expertise (see Marble and Boseovski, 2020). If positive information is inaccurate, children may need assistance to avoid inappropriate endorsement of incorrect information. Although this information may not reflect what they remember later, this initial endorsement could prompt repeated retrieval of inaccurate information, resulting in an illusion of truth (Dechêne et al., 2010).

Despite older children’s success on two sets of the knowledge boundary judgments, children across ages struggled to infer that both the zookeeper informant and the maternal figure could share general knowledge. Most children selected the maternal figure on more than half of the trials. One possible explanation for this pattern is that children were primed to think about the zookeeper informant and the maternal figure as members of distinct categories in a science center context. Children may have viewed “mothers” to be a broader category akin to “adults” but were not able to reflect that “zookeepers” could also be members of other categories. Accordingly, children did not generally endorse shared knowledge (i.e., an overlap in roles or identities) among these individuals. Indeed, even 6- to 8-year-olds treated “mothers” as more globally knowledgeable despite the option to select an answer choice of “both” on this assessment. This perception of maternal informants has implications for who children attend to during science center visits.

In general, it is somewhat surprising that a science center context did not prime children to prioritize information from the expert, zookeeper source. However, it is possible that children do not view their experiences at these locations as explicitly educational. Indeed, parents are sometimes less aware of the educational value of museum exhibits relative to the educators who organize these opportunities (e.g., Downey et al., 2010; but see Falk et al., 1998). If the entertainment value of science center experiences is emphasized (Rennie and McClafferty, 1995), children may be less likely to prioritize educational goals and pay attention to experts. Although science centers may face the unique challenge of increasing parent perceptions of experts as good sources of information (Luke et al., 2019), the findings from this study suggest that increasing the salience of expertise *via* clear labels or identification by a parent may promote children’s learning from these reliable sources (Gelman et al., 1998).

Indeed, this parental scaffolding may support learning when children miss cues to expertise or if speakers are prone to human fallibilities (e.g., poor explanations, Clegg et al., 2019; under-informativeness, Gweon et al., 2014). Elaborative conversations directed by caregivers have been an effective strategy to support the memory of younger children (Cleveland and Reese, 2005). Recent research suggests that parent-child conversations (e.g., Benjamin et al., 2010; Jant et al., 2014) and other memory developments (e.g., Pathman et al., 2011) figure prominently in what children remember from these autobiographical experiences, but these phenomena were not the focus of the present study. Parent-child conversations can also promote continued learning outside of museum settings and support children’s transfer of information from museum to home settings (e.g., Benjamin et al., 2010; Mills and Sands, 2020). The memory assessment in this study took place without the benefit of this scaffolding, which may partially explain why children only remembered a few facts. The memory assessment performance in this study suggests that in a naturalistic setting, children may benefit from a small amount of key information, which may also be advantageous for programming. For example, formal expert talks can be kept short to allow more informal engagement with visitors or more interactive opportunities for children, which in turn might also contribute to the richness of the information that children remember (e.g., Imuta et al., 2018). Specifically, it is possible that the surrounding environment (e.g., other visitor conversations, noises, and sights) affects children’s ability to focus explicitly on the target exhibit. Indeed, children’s ability to control their attention and ignore distractions improves across early and middle childhood (Best and Miller, 2010).

### Limitations and Future Directions

Some aspects of the method used in the current study may have limited children’s ability to remember information about the tamandua and highlight important considerations for comparisons between laboratory-based and naturalistic research. For example, the presentation method of information about the tamandua prioritized experimental control but as a result might not have followed a truly narrative format. Given that children’s recall is enhanced when an event follows a narrative structure (see Nelson and Fivush, 2004, for review), it is possible that children in this study would have benefitted from a more story-like presentation of facts. Another possibility is that recall would benefit from an exhibit that involved “hands-on” interaction (e.g., Imuta et al., 2018). In addition, the on-location memory assessment provided a compelling snapshot of children’s judgments and memory, but this procedure does not inform our understanding of children’s long-term memory for exhibit information. Nonetheless, a dissociation between what children endorsed as correct and what they remembered emerged, which presents compelling avenues for future research regarding children’s informal learning. Future research might also consider the inclusion of a source memory task to address questions regarding children’s encoding of information and should generally address how children’s priorities when they evaluate information map onto learning outcomes across a variety of contexts. It is likely that a combination of motivational biases in the moment and memory-specific effects play a role in these outcomes.

Another limitation of the current study could involve the differences between an experimental paradigm and expert behavior in naturalistic settings. Based on both formal and informal observations by the research team at this same science center, it appeared that the true experts engaged variably with both large groups and individual visitors, whereas real parents tended to address their individual children in conversation. In contrast, we sought experimental control for the possible effect of consensus or group effects and decided to retain the one-on-one element of the paradigm adapted from Boseovski and Thurman (2014), particularly because the “group” was composed of non-participating museum visitors. Future research might address the consensus element of naturalistic settings as well as the possibility that an expert and parent would engage in conversation with one another rather than taking individual turns to relay information.

### Conclusion

Taken together, the results from this study shed light on an important distinction between children’s acceptance of information during exhibit experiences and what they remember from these interactions. These findings demonstrate the strength of considering children’s developmental trajectories across multiple literatures to better understand children’s everyday learning in these prevalent naturalistic settings. These findings also highlight the need to extend research on children’s judgments of everyday expert and non-expert sources in naturalistic settings. Children’s sensitivity (or lack thereof) to who shares information and what those individuals say may enhance or hinder children’s learning outcomes at these important naturalistic locations.

## Data Availability Statement

The raw data supporting the conclusions of this article will be made available by the authors, without undue reservation.

## Ethics Statement

The studies involving human participants were reviewed and approved by The Office of Research Integrity at the University of North Carolina at Greensboro. Written informed consent to participate in this study was provided by the participants’ legal guardian/next of kin.

## Author Contributions

JB and TP conceptualized the design of this study. JB, TP, and SM supervised this project and provided guidance on data analyses and contributed to the writing and editing of this manuscript. KM, MS, JC, and KB collected the data or participated as the informants for this study. KM wrote the manuscript with contributions to the writing and feedback from JC and KB. All authors contributed to the article and approved the submitted version.

### Conflict of Interest

The authors declare that the research was conducted in the absence of any commercial or financial relationships that could be construed as a potential conflict of interest.
